# Olive oil intake is inversely related to cancer prevalence: a systematic review and a meta-analysis of 13800 patients and 23340 controls in 19 observational studies

**DOI:** 10.1186/1476-511X-10-127

**Published:** 2011-07-30

**Authors:** Theodora Psaltopoulou, Rena I Kosti, Dimitrios Haidopoulos, Meletios Dimopoulos, Demosthenes B Panagiotakos

**Affiliations:** 1 Department of Hygiene, Epidemiology and Medical Statistics, School of Medicine, University of Athens, M. Asias 75, Goudi, 11527, Athens, Greece; 2 Department of Obstetrics and Gynaecology, "Alexandra" Hospital, School of Medicine, University of Athens, Vas.Sofias 80, Goudi, 11528, Athens, Greece; 3 Department of Clinical Therapeutics, "Alexandra" Hospital, School of Medicine, University of Athens, Vas.Sofias 80, Goudi, 11528, Athens, Greece; 4 Department of Nutrition and Dietetics, Harokopio University, 70 Eleftheriou Venizelou str., Kallithea, 17671, Athens, Greece

**Keywords:** cancer, olive oil, Mediterranean diet, review, systematic, meta-analysis

## Abstract

Dietary fat, both in terms of quantity and quality, has been implicated to cancer development, either positively or negatively. The aim of this work was to evaluate whether olive oil or monounsaturated fat intake was associated with the development of cancer. A systematic search of relevant studies, published in English, between 1990 and March 1, 2011, was performed through a computer-assisted literature tool (i.e., Pubmed). In total 38 studies were initially allocated; of them 19 case-control studies were finally studied (13800 cancer patients and 23340 controls were included). Random effects meta-analysis was applied in order to evaluate the research hypothesis. It was found that compared with the lowest, the highest category of olive oil consumption was associated with lower odds of having any type of cancer (log odds ratio = -0.41, 95%CI -0.53, -0.29, Cohran's Q = 47.52, *p *= 0.0002, *I*-sq = 62%); the latter was irrespective of the country of origin (Mediterranean or non-Mediterranean). Moreover, olive oil consumption was associated with lower odds of developing breast cancer (logOR = -0,45 95%CI -0.78 to -0.12), and a cancer of the digestive system (logOR = -0,36 95%CI -0.50 to -0.21), compared with the lowest intake. The strength and consistency of the findings states a hypothesis about the protective role of olive oil intake on cancer risk. However, it is still unclear whether olive oil's monounsaturated fatty acid content or its antioxidant components are responsible for its beneficial effects.

## Introduction

Dietary fat, both in terms of quantity and quality, has been implicated to cancer development, either positively or negatively. Monounsaturated and polyunsaturated fats, deriving from olive oil and fish oil respectively, are among those that data are emerging for their relation to certain cancer types [1-3]. Concerning olive oil, no answer still exists as to whether the monounsaturated fatty acid content or the antioxidant components of its unsaponifiable fraction are responsible for its beneficial effects. Its fatty acid composition is mainly oleic acid, followed by palmitic and linoleic acids [4]. In addition, extra-virgin olive oil contains phenolic antioxidants, including simple phenols, aldehydic secoiridoids, flavonoids and lignans [5,6]. The high content of oleic acid makes olive oil far less susceptible to oxidation than the polyunsaturated fatty acids, for example. Also, olive oil's most representative phenols are thought to be potent scavengers of superoxide and other reactive species, a possible step for mutagenesis [7].

The effect of olive oil on human health has, till now, mainly been analyzed by studies deriving from Mediterranean populations, where it is consumed in large quantities. Almost all studies do not distinguish between 'plain' olive oil, which is the most used in the world market, and extra-virgin olive oil. Moreover, as it has been already stated, before causally interpreting the usually observed inverse association of olive oil to certain malignancies, residual confounding is an issue, as well as other limitations arising from the fact that associations are reported mainly from case-control studies [8].

To the best of our knowledge, there is no published systematic review and meta-analysis regarding the effect of olive oil consumption on cancer occurrence. Thus, the aim was to evaluate whether olive oil intake is associated with various types of cancer.

## Methods

### Studies selection and data extraction

Original research studies published in English between 1990 and March 1, 2011 were selected through a computer-assisted literature search (i.e., Pubmed, http://ncbi.nlm.nih.gov/PubMed). Combinations of key words relating to the aim of the paper were used, i.e. olive oil, Mediterranean diet, and cancer, or neoplasms. Also, specific searches are included: olive oil and, alternatively, prostate cancer, cancer of the larynx, oral carcinoma, cancer of the pharynx, cancer of the oesophagus, stomach cancer, lung cancer, ovarian cancer, renal cancer, endometrial cancer, pancreatic cancer, breast cancer, colorectal cancer, skin cancer, bladder cancer, haematological malignancies, leukaemia. In addition, the reference list of the retrieved articles was in some cases used to find articles not already allocated. The following information was abstracted according to a fixed protocol: study design, sample size, mean age and gender of participants, follow-up duration, assay methods, effect size measurements and potential confounders. The initial search (01.01.1990-01.03.2011, English language, humans) resulted in 248 entries on olive oil and cancer, 188 entries on olive oil and neoplasms, 297 entries on Mediterranean diet and cancer and 194 entries on Mediterranean diet and neoplasms. From the finally included papers, 3 were prospective, 35 were case-controls and none was a clinical trial. For the papers that they were not fully available in PubMed the information was retrieved from the abstract. This systematic review follows a cancer-specific structure; all papers referring to olive oil are included, and all papers referring to monounsaturated fats in Mediterranean countries are included too, because a substantial proportion of monounsaturated fat in traditional Mediterranean countries derives from olive oil. Finally, papers referring to the cancer risk examining the monounsaturated to saturated fats ratio were not taken into account, because this ratio is a proxy for olive oil intake, but not a definite evidence for its use and, thus, its beneficial effects. In addition, for the meta-analysis of the selected studies the MOOSE guidelines have been taken into account [9].

### Statistical analysis

Results of the studies are presented as odds ratios and their corresponding standard errors (SE) or 95% confidence intervals. From the finally included 38 papers (3 were prospective and 35 were case-controls), the meta-analysis was focused only on studies (n = 19) that solely evaluated raw olive oil intake, in order to eliminate the potential synergistic effect of cooking. Random effects meta-analysis of the selected studies was applied. The effect size measures used for the meta-analysis were the log-odds ratios (and the corresponding SE) of olive oil intake (highest vs. lowest available category) and the endpoints of the selected studies were cancers of above mentioned specified topographies. Heterogeneity was assessed using Cochran's Q and I^2 ^(I^2 ^ranges between 0% and 100%, with lower values representing less heterogeneity) and evaluated using the chi-squared test. Sensitivity analyses were performed according to some characteristics of the studies, such as: country of origin (Mediterranean or not) and type of cancer. To assess the presence of publication bias, the "funnel's plot" was tested. All statistical calculations were performed using NCSS 2004 software (Number Cruncher Statistical Systems Co., Utah, USA).

## Results

Table 1 summarizes studies' characteristics. The majority of the studies that completed the entry criteria for this review were case - control (i.e., 35), while only three were prospective.

**Table 1:** A summary of the selected studies according to the year published, design, region, follow-up, sample's characteristics and assay methods used.

Author	Year published	Design, region and follow-up duration	Sample size; mean age and gender; histological evaluation	Assay methods
***Breast cancer***				
Trichopoulou A, et al [10]	1995	Case-control study conducted in Greece from 1989 to 1991	820 women with breast cancer and 1548 controls	Olive oil intake more than once a day versus once a day. A semiquantitative questionnaire is used.
Katsouyanni K, et al [11]	1994	Hospital-based case-control study conducted in Greece from 1989 to 1991	820 patients with histological confirmed cancer of the breast were compared with 795 orthopaedic patient controls and 753 hospital visitor controls	Diet was ascertained through a semi-quantitative food-frequency questionnaire; quintiles of monounsaturated fat were measured.
Trichopoulou A, et al [12]	2010	Prospective study which evaluated the relation of conformity to the Mediterranean diet with breast cancer risk in the context of the European Prospective Investigation into Cancer and Nutrition cohort in Greece	14,807 women were followed up for an average of 9.8 y and identified 240 incident breast cancer cases	Diet was assessed through a validated food-frequency questionnaire, and conformity to the Mediterranean diet was evaluated through a score (range = 0-9 points) incorporating the characteristics of this diet. Among others, intake of olive oil and monounsaturated lipids were measured.
Martin-Moreno JM, et al [13]	1994	Population-based case-control study conducted in Spain	762 patients (18-75 years of age) and 988 female controls	Quartiles of olive oil consumption.
Landa MC, et al [14]	1994	Case-control study conducted in north Spain from 1988 to 1991	100 women with breast cancer and 100 hospital controls	Tertile of monounsaturated fat intake were measured.
García-Segovia P, et al [15]	2006	Case-control study conducted in Spain from 1999 to 2001	755 women: 291 incident cases with confirmed breast cancer and 464 controls randomly selected from the Canary Island Nutrition Survey	A semi-quantitative food-frequency questionnaire was completed; intake of monounsaturated fat and olive oil were measured.
La Vecchia C, et al. [16]	1995	Multicenter case-control study conducted between 1991 and 1994 in Italy	2564 histological confirmed patients and 2588 controls (aged 34-70 years)	Use of a validated food-frequency questionnaire; olive oil was measured.
Sieri S, et al [17]	2004	Prospective study conducted in northern Italy where women volunteers were recruited from 1987 to 1992 and were followed for 9.5 years	8,984 women were followed up for an average of 9.5 y, and identified 207 incident breast cancer cases	A semiquantitative food frequency questionnaire was used for the evaluation of four dietary patterns. Olive oil consumption was assessed in the salad vegetables pattern.
Bessaoud F, et al [18]	2008	Case-control study conducted in southern France (June 2002 to December 2004)	437 histological confirmed patients and 922 controls females, aged 25 to 85 years	Olive oil intake was assessed through a validated food-frequency questionnaire.
Richardson S, et al [20]	1991	Hospital-based case-control study conducted in France	409 patients and 515 controls	Tertile of consumption of monounsaturated fat intake was measured.
***Colorectal cancer***				
Braga C, et al [25]	1998	Multicenter case-control study in six Italian areas from 1992 to 1996	1953 patients with histological confirmed colorectal carcinoma (1225 of the colon and 728 of the rectum) (median age 62 years, range 23-74). Controls were 4154 subjects with no history of cancer (median age 58 years, range 20-74).	Tertiles of olive oil intake were measured. Olive oil intake was assessed through a food-frequency questionnaire including 78 foods, groups of foods or recipes.
Benito E, et al [29]	1991	Multicenter case-control study of colorectal cancer conducted in Spain from 1984 to 1988	286 colorectal cancer cases, 295 population controls and 203 hospital controls	Different monounsaturated fat intakes were measured. Food composition tables and ad-hoc estimates of portion sizes were used to derive intake estimates of 29 nutrients and of total calories.
Galeone C, et al [30]	2007	Multicenter case-control study of colorectal cancer conducted in Italy and Switzerland from 1992 to 2000	1394 cases of colon cancer (median 62 years), 886 cases of rectal cancer (median 63 years) and 4765 controls (median 58 years)	Use of fried olive oil was measured.
***Prostate cancer***				
Tzonou A, et al [34]	1999	Case-control study conducted from 1994 to 1997 in Greece	320 patients with histological confirmed prostate cancer and 246 controls (aged 71 years and 70 years, respectively)	Olive oil and other fat were measured. The food-frequency questionnaire comprised around 120 food items or beverages categories.
Norrish AE, et al [35]	2000	Population-based case-control study conducted in New Zealand from 1996 to 1997	317 prostate cancer cases and 480 controls from 40 to 80 years old	Quantiles of monounsaturated fat-rich vegetable oil consumption were measured.
Hodge A, et al [36]	2004	Population-based case-control study, where eligible cases were diagnosed between 1994 and 1997 in Australia	858 men aged < 70 years with histological confirmed cancer and 905 age-frequency-matched men, selected at random from the electoral rolls	Various olive oil intakes were measured. Food-frequency questionnaire had 121-items.
***Cancer of the larynx***				
Gallus S, et al [40]	2003	Combined dataset from two case-control studies conducted from 1986 to 2000 in northern Italy and Switzerland	68 women under age 79 years, with incident, histological confirmed cancer of the larynx (median age 60 years). Controls were 340 women, admitted to the same network of hospitals (median age 60 years)	Intake of olive oil was measured. Validated food-frequency questionnaire based on 78 foods or groups of foods was applied.
Crosignani P, et al [41]	1996	Prospective study to evaluate survival for laryngeal cancer cases interviewed 10 years ago in a population-based case-control study	213 incident cases of laryngeal cancer	Olive oil and other fat were measured through a food frequency questionnaire.
Bosetti C, et al [43]	2002	Case-control study conducted in Nothern Italy and the Swiss Canton of Vaud from 1992 to 2000	527 histological confirmed cases and 1297 frequency-matched controls	Dietary intakes 2 years prior to cancer diagnosis were estimated through a food-frequency questionnaire including 78 foods and beverages. Olive oil consumption was measured.
***Cancer of the oral cavity and pharynx***				
Lagiou P, et al [44]	2009	Multicenter case-control study in 14 centers in 10 countries, 2002 to 2005 in all centers but Paris (1987 to 1992)	1861 men and 443 women histological confirmed cancer patients and 1661 men and 566 women controls that were frequency-matched to patients by sex, and 5-year groups.	Olive oil consumption was recorded through a semi-quantitative food frequency questionnaire, specifically developed for ARCAGE. Olive oil was not recorded in the Paris center.
Franceschi S et al [46]	1999	Multicenter case-control in Italy carried out in 1992 to 1997	512 men and 86 women oral cavity and pharynx cancer cases (median age 58, range 22-77) and 1008 men and 483 women controls (median age 57, range 20-78)	Food-frequency questionnaire included 78 foods, food groups or recipes, including olive oil intake.
Nešić V, et al [47]	2010	Case-control study conducted in Belgrade of Serbia, during the period 2001-2003	45 cases with histopathological diagnosis of UCNT and 90 controls that were matched by sex, age (± 3 years), and place of residence (city-village)	Dietary data were collected using a food frequency questionnaire (FFQ) included 100 food items. Olive oil consumption was measured.
Petridou E, et al [48]	2002	Hospital-based case-control study in Greece	106 patients and 106 control subjects.	Different intakes of added lipids (olive oil is a substantial fraction) were measured. Dietary intake was assessed through a validated, semi-quantitative food-frequency questionnaire.
***Cancer of the oesophagus***				
Tzonou A, et al [50]	1996	Hospital-based case-control study conducted in Greece from 1989 to 1991	99 patients (43 patients with incident esophageal squamous-cell carcinoma and 56 patients with incident esophageal adenocarcinoma) and 200 controls	The frequency of intake of monounsaturated fats was measured. Diet was assessed through a semiquantitative food-frequency questionnaire.
Bosetti C, et al [51]	2000	Multicenter case-control study in 3 areas of northern Italy from 1992 to 1997	304 patients (275 men, 29 women) (median age 60, range 39-77) and 743 controls (593 men, 150 women) (median age 60, range 36-77)	Olive oil intake was measured through a food-frequency questionnaire that included 78 specific foods and beverages.
Launoy G, et al [53]	1998	Multicenter case-control study conducted between 1991 and 1994 in France	208 histological confirmed patients and 399 controls, all males	Different types of olive oil intake were measured through a standardized detailed food questionnaire about the previous year's diet.
***Stomach cancer***				
Palli D, et al [54]	2001	Population-based case-control study in Italy conducted between 1985-1987	126 patients with MSI status (MSI + = 43, MSI- = 83) and 561 controls	Tertiles of olive oil consumption and other lipids were measured.
***Lung cancer***				
Fortes C, et al [56]	2003	Hospital-based case-control study conducted in Italy from1993 to 1996	Cases were 342 patients with newly diagnosed primary lung cancer and controls were 292 adults (all aged more than 35 years)	Olive oil intake was measured.
***Ovarian cancer***				
Tzonou A, et al [60]	1993	Hospital-based case-control study conducted in Greece from 1989 to 1991	189 patients and 200 controls under 75 years of age	Monounsaturated fat and other lipids were measured.
Bosetti C, et al [61]	2002	Multicenter case-control study, conducted from January 1992 to September 1999	1031 histological confirmed patients (median age 56, age range 18-79 years) and 2411 hospital controls (median age 57, age range 17-79 years).	Seasonal lipid consumption, such as olive oil and other fats were measured. The specific food-frequency questionnaire included 78 specific foods and beverages.
***Endometrial cancer***				
Levi F, et al [65]	1993	Case-control study conducted in Switzerland and northern Italy	274 histological confirmed patients and 572 controls	Olive oil intake was measured. Diet was assessed using a questionnaire which considered 50 indicator foods, including the major sources of energy.
Tzonou A, et al [66]	1996	Hospital-based case-control study undertaken in Greece from 1992 to 1994	145 histological confirmed patients and 298 controls	Intake of monounsaturated fat, mostly olive oil was measured.
Petridou E, et al [67]	2002	Hospital-based case-control study undertaken in Greece	84 histological confirmed patients and 84 controls with intact uterus	Olive oil was measured.
***Pancreatic cancer***				
Kalapothaki V, et al [68]	1993	Hospital-based case-control study conducted in Athens from 1991 to 1992	181 cases and 181 hospital-181 hospital visitor controls	Monounsaturated fat was measured. Food-frequency questionnaire was assessing the consumption of 110 food items or beverages over the period of one year before the onset of the disease.
Soler M, et al [69]	1998	Case-control study conducted in Italy between 1983 and 1985	362 patients with histological confirmed cancers of the pancreas and 1552 controls	Tertiles of olive oil intake were measured.
La Vecchia and Negri [70]	1997	Case-control study conducted in Italy between 1983 and 1985	362 patients with histological confirmed cancers of the pancreas and 1502 controls	Tertiles of olive oil intake were measured.
***Bladder cancer***				
Brinkman MT et al [74]	2011	Case-control study conducted in Belgium	200 cases and 386 controls	Tertiles of olive oil intake were measured.
Riboli E et al [75]	1991	Multi-centre case-control study conducted in Spain	432 male cases and 792 age matched controls	Monounsaturated fat intake was measured. Usual dietary habits were investigated by means of an interview-based dietary history questionnaire.

In Table 2 the main findings of each study are summarised.

**Table 2:** Effect size measures and confounding factors used in the selected studies that were included in the systematic review.

Author	Effect sizes (OR = odds ratio)	95% Confidence Interval	Confounding factors
***Breast cancer***			
Trichopoulou A, et al [10]	Increased olive oil consumption is related with reduced cancer risk (OR = 0.75 for more than once a day versus once a day)	0.57-0.98	Adjustment for age, place of birth, parity, age at first pregnancy, age at menarche, menopausal status, Quetelet index, total energy intake, consumption of fruits and vegetables
Katsouyanni K, et al [11]	OR per quintile monounsaturated fat 0.97	0.88-1.07	Adjustment for demographic and reproductive risk factors for breast cancer, as well as for total energy intake and mutual confounding influences among nutrients
Trichopoulou A, et al [12]	HR per 21 g in daily intakes of olive oil in the entire cohort (HR = 0.93, P = 0.106) and in the postmenopausal women (HR = 0.85, P = 0.106)	0.80-1.08 0.69-1.06	Adjusted for age, educational level, smoking status, BMI, height (ordered as quintiles), metabolic equivalents of task hours per day, energy intake, age of menarche, parity, age at first delivery, menopausal status, age at menopause, hormone replacement therapy and an interaction term for the BMI by menopausal status.
Martin-Moreno JM, et al [13]	For highest versus lowest quartile of olive oil consumption, OR = 0.66	0.46-0.97	Adjustment for total energy intake and other potential confounders
Landa MC, et al [14]	OR for the highest tertile of monounsaturated fat intake compared to the lowest 0.30	0.1-1.08	Not mentioned in the abstract in PubMed
García-Segovia P, et al [15]	The OR for women in the three upper quintiles of olive oil consumption (≥ 8.8 g/day) is 0.27 OR for monounsaturated fat intake is 0.52	0.17-0.42 0.30-0.92	Not mentioned in the abstract in PubMed
La Vecchia C, et al [16]	OR per unit (30 g) is 0.89. The ORs for olive oil compared with the lowest intake are 1.05, 0.99, 0.93 and 0.87 for increasing quintiles of intake.	0.81-0.99	Adjusted for demographic and reproductive breast-cancer risk factors, energy intake and mutually for types of dietary fat
Sieri S, et al [17]	The salad vegetables pattern had a RR = 0.66	0.47-0.95	Adjusted for education, parity, height, age at menarche, smoking, menopausal status, energy intake and age
Bessaoud F, et al [18]	OR (> 20.5 g/day vs. < 2 g/day) 0.71 (classical method) OR (> 20.5 g/day vs. < 2 g/day) 0.29 (spline method)	0.44-1.14 0.18-0.47	Adjustment for total energy intake, education, parity, breast-feeding age at first full-term pregnancy, duration of ovulatory activity, body mass index, physical activity, and first-degree family history of breast cancer. Adjustment by monounsaturated fatty acids and total energy intake
Richardson S, et al [20]	OR for the highest tertile of consumption of mono-unsaturated fat = 1.7	1.2-2.5	Not mentioned in the abstract in PubMed
***Colorectal cancer***			
Braga C, et al [25]	ORs for the highest tertile of olive oil intake, compared with the lowest one is 0.83 when colorectal carcinoma is analyzed as a whole/0.81 for colon carcinoma and 0.88 for rectal carcinoma ORs are 0.94 for colorectal 0.94 for colon carcinoma 0.97 for rectal carcinoma	0.70-0.99 0.66-0.99 0.66-1.12 0.79-1.12 0.76-1.16 0.75-1.25	Estimates from multiple logistic regression equations are presented, including terms for study center, age, sex, education, alcohol, total energy intake, and simultaneously the various types of oils and fats. After allowance for vegetable intake.
Benito E, et al [29]	ORs for the higher available category of monounsaturated fat intake compared for the lowest one is 0.72	Not mentioned in the abstract in PubMed	Adjustment for total calorie intake
Galeone C, et al [30]	OR for fried olive oil, 0.89, for colon cancer	0.82-0.98	Adjusted for age, center, sex, education, body mass index, tobacco smoking, alcohol drinking, non alcohol energy intake, family history, physical activity and red meat intake.
***Prostate cancer***			
Tzonou A, et al [34]	Chi-square linear trend adjusted = 0.44		Adjusted for age, height, Quetelet index, years of schooling and total energy intake.
Norrish AE, et al [35]	RR 0.5 (> 5.5 ml MUFA-rich vegetable oil intake per day vs. non-consumption)	0.3-0.9	The multivariate linear regression model included terms for age, total non-steroidal anti-inflammatory drugs, socioeconomic status, intake of total energy, lycopene, and levels of eicosapentaenoic acid and docosahexaenoic acid measured in erythrocytes
Hodge A, et al [36]	Higher consumption of olive oil (> 0.25, as well as < 0.25 l/month compared to non consumption) had an OR = 0.8	0.6-1.1	Adjusted for state, age group, year, country of birth, socioeconomic group, total energy intake and family history of prostate cancer
***Cancer of the larynx***			
Gallus S, et al [40]	OR for the olive oil higher intake compared to the lower one was 0.28	0.09-0.89	Adjusted for age, year of interview and study center, and including terms for education, BMI, non-alcohol energy intake, tobacco and alcohol consumption
Crosignani P, et al [41]	The consumption of olive oil was associated with a better prognosis from laryngeal cancer	Not mentioned in the abstract in PubMed	Not mentioned in the abstract in PubMed
Bosetti C, et al [43]	OR = 0.4 for the highest compared to the lowest quintile When adjusted for total vegetable consumption: OR = 0.66	0.3-0.7 0.39-1.09	Estimates from unconditional logistic regression adjusted for sex, age, center, education, tobacco smoking, alcohol drinking, non-alcohol energy intake, all seasoning fats in the table, as well as for total vegetable consumption in the second model.
***Cancer of the oral cavity and pharynx***			
Lagiou P, et al [44]	Center specific median was used as a cut-off. For olive oil above versus below median: Olive oil (overall) OR 0.78 Olive oil in salads OR 0.84 Olive oil for cooking OR 0.65	0.67-0.90 0.70-1.00 0.55-0.78	Adjusted for centre through stratification and controlled for age, gender, BMI, height, education level, alcohol consumption and smoking status.
Franceschi S et al [46]	Olive oil OR = 0.4 (highest vs. lowest quintile) Vegetable-adjusted OR = 0.6 for the same quintiles	0.3-0.7 0.4-0.9	Adjusted for age, centre, sex, education, smoking habit, total intake of alcohol and energy, plus all oils and fats examined.
Nešić V, et al [47]	For frequent/moderate consumption vs. rare or never, OR 0.42	0.19-0.91	Variables, which were significantly associated with nasopharyngeal cancer in each of the multivariate analyses, were included in the final model (consumption frequency of eggs, margarine, olive oil, rice, white bread, cornbread, peanuts and industrially manufactured food additives for enhancing flavour, as well as "passive smoking" in the family during childhood, chronic rhinosinusitis and positive family history for malignant tumours outside of the otorhinolaryngology region)
Petridou E, et al [48]	Added lipids, which in Greece are overwhelmingly olive oil, OR = 0.75 (per quantile of intake)	0.58-0.99	Adjusted for body mass index, height, years of schooling, condition of teeth, energy intake, tobacco smoking, daily alcohol and coffee consumption and total energy intake.
***Cancer of the oesophagus***			
Tzonou A, et al. [50]	OR associated with an increment of a marginal quintile in the frequency of intake of monounsaturated fat is 1.07 for adenocarcinoma of the oesophagus. The respective OR is 0.74 for squamous cell carcinoma	0.72-1.60 0.49-1.11	Adjusted for socio-demographic facts, tobacco smoking, consumption of alcoholic beverages and total energy intake
Bosetti C, et al. [51]	OR = 0.36 for the highest compared to the lowest quintile	0.18-0.73	Adjusted for age, sex, area of residence, education, tobacco smoking, alcohol drinking, non-alcohol energy, all added lipids and for total energy consumption
Launoy G, et al [53]	OR for consumers versus non-consumers 0.70	0.54-0.90	Adjusted for age, interviewer, smoking, beer, aniseed aperitifs, hot Calvados, whisky, total alcohol, total energy intake and other food groups.
***Stomach cancer***			
Palli D, et al [54]	OR = 0.6, for the highest versus the lowest tertile (MSI-) OR = 0.5, for the highest versus the lowest tertile (MSI+)	0.3-1.00 0.2-1.1	Adjusted for age, sex, social class, family history of gastric cancer, area of residence, and BMI tertiles, and total energy.
***Lung cancer***			
Fortes C, et al [56]	Exclusive use of olive oil OR = 0.67	0.45-0.99	Adjusted for smoking variables and also considering all food items simultaneously
***Ovarian cancer***			
Tzonou A, et al [60]	The adjusted OR associated with an increment of about 1 SD of the energy-adjusted residual of monounsaturated fat was 0.80	0.65-0.99	Adjusted for age, years of schooling, parity, age at first birth, menopausal status, as well as for energy intake and other nutrients in the same model, such as crude fiber.
Bosetti C, et al [61]	A reduced risk of ovarian cancer was observed for the highest quintile of olive oil OR = 0.68 compared to the lowest one. OR were 0.85 for olive oil per 12 gr. Allowance for total vegetable intake attenuated the effect OR = 0.82	0.50-0.93 0.76-0.95 0.60-1.14	Adjusted for study centre, year at interview, age, education, parity, oral contraceptive use, and total energy intake, various types of added oils and fats simultaneously, plus total vegetable intake, when indicated.
***Endometrial cancer***			
Levi F, et al [65]	OR for the highest versus the lowest tertile of olive oil intake OR = 0.82	Not mentioned in the abstract in PubMed	Not mentioned in the abstract in PubMed
Tzonou A, et al [66]	Increasing intake of monounsaturated fat, mostly olive oil, by about one standard deviation was associated with a OR = 0.74	0.54-1.03	Adjusted for age, schooling years, age at menopause, number of liveborn children, number of miscarriages, number of abortions, history of use of menopausal estrogens, smoking, alcohol intake, coffee drinking, height, body mass index and energy intake, as well as for protein, saturated and polyunsaturated fat
Petridou E, et al [67]	Highly suggestive protective effect of added lipids, which in the Greek diet are primarily represented by olive oil	Not mentioned in the abstract in PubMed	Not mentioned in the abstract in PubMed
***Pancreatic cancer***			
Kalapothaki V, et al [68]	OR = 1.04 (hospital controls) OR = 0.97 (visitor controls) These are associated with an increment of about one standard deviation of the energy-adjusted residual of monounsaturated fat intake	0.86-1.25 0.80-1.17	Controlling for age, gender, hospital, past residence, years of schooling, cigarette smoking, diabetes mellitus and energy intake
Soler M et al [69]	OR = 0.58	0.35-0.97	Adjusted for socio-demographic factors and smoking
La Vecchia and Negri [70]	OR 0.76 for the intermediate OR 0.60 for the highest score of intake	Not mentioned in the abstract available in Pubmed	Not mentioned in the abstract in Pubmed
***Bladder cancer***			
Brinkman MT, et al [74]	Comparing the highest with the lowest tertiles of olive oil intake between cases and controls using unconditional logistic regression. Middle versus the lowest tertile (OR: 0.62; and the highest versus the lowest tertile (OR: 0.47, p-trend = 0.002)	0.39-0.99 0.28-0.78	Adjustment was made for age, sex, smoking characteristics, occupational exposures and calorie intake
Riboli E et al [75]	Moderate increases in the risk for higher intake of monounsaturated fat were found, which disappeared after correction for saturated fat	Not mentioned in the abstract in PubMed	Adjusted for tobacco smoking and energy intake.

### Breast cancer

Several case-control studies, conducted in Greece, Spain, Italy and France suggest that olive oil may be associated with decreased breast cancer. In a Greek hospital-based case-control study of 820 women with breast cancer and 1548 controls, after adjustment for energy intake, more frequent consumption of olive oil was associated with significantly reduced breast cancer risk (odds ratio, OR = 0.75) for more than once a day compared with once a day. This protective association was concentrated in postmenopausal women, but the relevant interaction term was not statistically significant [10]. Evaluating specifically the relation of monounsaturated fat intake with breast cancer, no suggestive association was shown (mutually adjusted RR per quintile 0.97, 95%CI 0.88 to 1.07) [11]. Furthermore in Greece, Trichopoulou et al. [12] studied the relation of conformity to the Mediterranean diet with breast cancer risk in the context of the European Prospective Investigation into Cancer and Nutrition cohort in Greece. Fourteen thousands eight hundred women were followed up for an average of 9.8 years and identified 240 incident breast cancer cases. Results showed that although increasing conformity to the Mediterranean diet was not associated with lower breast cancer risk in the entire cohort, however there was a marginally significant inverse association among postmenopausal women (HR = 0.78 for every 2 points; 95%CI 0.62 to 0.98). Moreover, results showed that the increased consumption of olive oil was associated with lower breast cancer risk in the entire cohort (HR = 0.93, 95%CI 0.80 to 1.08) and in the postmenopausal women (HR = 0.85, 95%CI 0.69 to 1.06) respectively, though not statistically significant [12].

Three case-control studies derive from Spain; in the first one (762 cases - 988 controls), for olive intake, the odds ratios for the highest versus the lowest quartile of consumption was 0.66, with a significant dose-response trend [13]. In the second (100 breast cancer cases - 100 controls), women in the highest tertile of monounsaturated fat consumption were at lower risk compared with women in the lowest tertile (RR = 0.30; 95%CI 0.1 to 1.08) [14]. At last, in the Canary Islands study, the odds ratio for women in the three upper quintiles of olive oil consumption (≥ 8.8 g/day) was 0.27 [15]. In Italy, in a multicenter case-control study (2564 cases - 2588 controls), the odds ratio per unit (30 g) increase of olive oil consumption was 0.89 (95%CI 0.81 to 0.99); the ORs for increasing levels of olive oil consumption, adjusted for total calories, were found as 1.05, 0.99, 0.93 and 0.87 [16]. In the same country, in a prospective study that used nutritional data from 8984 women, an average follow-up of 9.5 years and 207 incident cases of breast cancer, the participants completed a semiquantitative FFQ, during 1987 to 1992 (ORDET cohort). The "salad vegetables" pattern, principally consisting of raw vegetables and olive oil, was associated with significantly lower (34-35%) breast cancer incidence (RR = 0.66, 95%CI 0.47 to 0.95), comparing the highest to lowest tertile [17]. In south France, a case-control study of 437 breast cancer cases and 922 controls showed an inverse association of olive oil intake with breast cancer risk [18].

In France, in a prospective study, Cottet et al. [19] examined the association between dietary patterns and breast cancer risk. The analyses included 2,381 postmenopausal invasive breast cancer cases diagnosed during a median 9.7-year follow-up period (1993-2005) among 65,374 women from the E3N-EPIC cohort. Results showed that the "healthy/Mediterranean" pattern was negatively associated with breast cancer risk (hazard ratio = 0.85, 95%CI 0.75 to 0.95; P = 0.003 for linear trend), especially when tumors were estrogen receptor-positive/progesterone receptor-negative. Authors concluded that adherence to a dietary pattern including mostly fruits, vegetables, fish, and olive/sunflower oil, along with avoidance of Western-type foods, could result in a substantial reduction in postmenopausal breast cancer risk [19]. Finally, in France, in another study, a positive association of monounsaturated fat intake with breast cancer risk was found [20].

Another study, the EURAMIC study, used adipose biopsies with diverse fat intake patterns from 5 European centres, including southern Europe (Malaga, Spain), to see if oleic acid or other monounsaturates are associated with breast cancer. In 291 postmenopausal incident breast cancer patients and 351 controls the OR (75^th ^to 25^th ^percentiles) was 0.40 in Malaga and 1.27 (not statistically significant) in all the other centres pooled. Thus, the strong inverse association between oleic acid concentrations and breast cancer in the Spanish study population was not observed in the study's non-Spanish populations [21].

### Colorectal cancer

A number of case-control and prospective studies indicate that there is no appreciable association between intake of total, saturated, monounsaturated, or polyunsaturated fat intakes, in general, with the risk of colorectal cancer, or that the existing results are unconvincing [22-24].

Examining the association of olive oil and monounsaturated fat intake mainly in Mediterranean populations, with colorectal cancer in case-control studies, olive oil has been found to have a slight protective effect, while monounsaturated fat intake appeared uninfluential. In an Italian study of 1953 patients with colorectal carcinoma (1225 colon and 728 rectum) and 4154 controls, the ORs for successive tertiles, compared with the lowest one, were 0.87 (95%CI 0.75 to 1.01) and 0.83 (95%CI 0.70 to 0.99) (p = 0.03) when colorectal carcinoma was analyzed as a whole, 0.82 (95%CI 0.68 to 0.98) and 0.81 (95%CI, 0.66 to 0.99) (p = 0.04) for colon carcinoma, and 0.96 (95%CI 0.77 to 1.19) and 0.88 (95%CI 0.66 to 1.12) for rectal carcinoma. After allowance for vegetable intake, results were in the same direction, not statistically significant [25]. In the same sample [26,27] monounsaturated fat intakes appeared uninfluential [28]. Results towards the same direction were presented by Benito et al. [29] in 1991 [4,29]. When, patients from Switzerland were examined together (recruited from 1992 to 2000, 1394 colon cancer cases, 886 rectal cancer cases and 4765 controls), the OR for an increment of one portion of fried foods per week was 0.89 for colon cancer, 0.97 for rectum and 0.93 for colorectum, for the use of olive oil [30].

Other studies examining the role of monounsaturated fat in colorectal cancer outside of the Mediterranean basin were not taken into consideration, because in these countries monounsaturated fat may derive from sources other than olive oil [31-33].

### Prostate cancer

In three case-control studies, neither monounsaturated lipids deriving mainly from olive oil nor olive oil per se were found to be associated with prostate cancer risk. In a case-control study in Greece, 320 patients with histologically confirmed incident prostate cancer and 246 controls were included [34]. Among added lipids, after adjustment for total energy intake among others, olive oil was unrelated to the risk (p = 0.66), though monounsaturated fats, largely deriving from meat or olive oil, were positively associated (OR 1.05, 95%CI 0.75 to 1.48, p = 0.77). As it is stated, this contradiction could in part be explained by the high content of vitamin E in olive oil, which was significantly inversely related to prostate cancer risk (OR 0.53, 95%CI 0.30 to 0.94, p = 0.03). In another population-based case-control study in New Zealand, after adjusting for energy intake, 317 prostate cancer cases and 480 controls completed a food-frequency questionnaire, in which for monounsaturated fatty acids-rich vegetable oils, a single question was asked about usual consumption of olive oil, canola or peanut oil, collectively, whereas biomarkers for fatty acids were measured in erythrocytes [35]. Increased consumption of MUFA-rich vegetables oils (not total MUFA, or MUFA of animal origin) was inversely associated with prostate cancer risk. Thus, according to the authors, a specific cancer-protective effect could be attributed to non-MUFA components of vegetable oils, as the antioxidant components of olive oil. In another population-based case-control study in Australia in 858 men aged (< 70 years) with histologically confirmed prostate cancer (Gleason grade > 5) and 905 age-frequency-matched men, higher consumption of olive oil (> 0.25 and < 0.25 l/month compared to non-consumption) had an OR = 0.8 (p for trend = 0.12) [36]. There was evidence for an inverse association of oleic acid with prostate risk with a marginal non-significance, which seems difficult to interpret, because olive oil only supplied 8% of oleic acid consumed.

Monounsaturated fat intakes and their association with prostate cancer in different countries than Mediterranean were not taken into consideration, since a major source of monounsaturated fat in North America, for example, is meat [37-39].

### Cancer of the larynx

Concerning laryngeal cancer, a protective effect of olive oil intake was found in cross-sectional studies. An analysis was performed in the combined dataset from two case-control studies (1986-2000) conducted in northern Italy and Switzerland for 68 women less than 79 years, with histologically confirmed cancer of the larynx and 340 controls. After multivariate adjustment, an inverse association was found between high versus low intake of olive oil and laryngeal cancer (OR = 0.28, 95% CI 0.09 to 0.89) [40]. Olive oil has been related to a better prognosis to male laryngeal cancer patients, whereas, the same study group published a study in which the occurrence of new primaries among male laryngeal cancer patients was lowered by one-third with higher intake of monounsaturated fatty acids [41,42]. In another study conducted in the same countries, with 527 histologically confirmed laryngeal cancer cases and 1297 controls, an inverse association of the risk was observed with olive oil (OR = 0.4 for the highest compared to the lowest quintile of intake, p = 0.003), not statistically significant when controlling for total vegetable consumption (OR = 0.66, p = 0.45) [43]. Finally, in an analysis where cancers of the oral cavity, pharynx other than nasopharynx, larynx and oesophagus were collectively assessed [44], olive oil used in salads and/or in cooking, was significantly inversely associated (OR contrasting frequency of consumption above versus below median = 0.78 with 95%CI 0.67 to 0.90).

### Cancer of the pharynx and the oral cavity

As it is stated in the paper by Garavello et al. [45] analysing the few prospective and case-control studies available, monounsaturated fats (and olive oil) seem to be inversely related to oral and pharyngeal cancer risk. In a case-control analysis in Italy (1992-1997), with 598 cases and 1491 controls, risk was approximately halved in the highest compared to the lowest quintile of olive oil (OR = 0.4, 95%CI 0.3 to 0.7), which was slightly attenuated by allowance for vegetable intake [46]. Moreover, in a recently conducted case-control study in Servia which included 45 cases with histopathological diagnosis of undifferentiated carcinoma of nasopharyngeal type (UCNT), and 90 controls, frequent/moderate consumption of olive oil was significantly negatively associated with UCNT compared with rare or never consumption of olive oil (OR = 0.42, 95%CI 0.19 to 0.91, p = 0.03) [47].

For oral cancer exclusively, in a case-control study in Greece with 106 patients with oral carcinoma and an equal number of controls, added lipids, which in Greece are represented overwhelmingly with olive oil, were found inversely significantly associated with oral carcinoma risk (p = 0.04) [48]. The joint analysis of Lagiou et al. [44], for oral, pharyngeal, laryngeal and oesophageal carcinoma, confirmed the same results. Finally, in a recently conducted case-control study in Greece in the context of the European alcohol-related cancers and genetic susceptibility in Europe project (239 incident upper aerodigestive tract, UADT, cases and 194 hospital controls), authors concluded that stricter adherence to the Mediterranean diet was associated with a substantial and significant decrease in UADT cancer risk (30% for a two-unit increase in score), whereas after mutual adjustment, no individual dietary component of this diet was significantly associated with this risk [49].

### Oesophageal cancer

In three case-control studies, one yields inconclusive results for the association of monounsaturated lipids with oesophageal cancer and two others show a significant inverse association with olive oil intake. In Greece, 56 cases of adenocarcinoma and 43 cases of squamous cell carcinoma were compared with 200 controls. Neither for adenocarcinoma (OR = 1.07, 95%CI 0.72 to 1.60 between an increment of a marginal quintile in the intake of monounsaturated lipids), nor for squamous cell carcinoma (OR = 0.74, 95%CI 0.49 to 1.11 for the same increment), an association with monounsaturated lipids (mainly olive oil) was established [50,4].

In the second study (1992 to 1997) in northern Italy, 304 histologically confirmed squamous oesophageal carcinoma cases and 743 controls were interviewed, after correction for multiple confounders, olive oil intake showed a significant reduction of cancer risk (OR = 0.4). Without adjustment for total vegetable consumption, olive oil also showed a significant reduction of cancer risk, with no monotonic exposure association, that is, even consumers of a minimal quantity of olive oil appeared to be at a reduced risk for the disease, as consumers of a maximal quantity (OR = 0.3, 95% CI = 0.1 to 0.5), for the highest versus the lowest quintile of olive oil intake [51] and the same significant reduction was also found for monounsaturated fatty acids [52]. In a third case-control study in France (208 cases and 399 controls), all males, for olive oil, the OR for consumers versus non-consumers was 0.70 (95% CI 0.54 to 0.90) [53].

### Stomach cancer

For stomach cancer, in Italy, 83 stomach cancer cases of negative microsatellite instability (MSI) (a distinctive molecular pathway of carcinogenesis) were dietary assessed. Olive oil had a statistically significant protective effect (OR = 0.6, 95%CI 0.3 to 1.00, for the highest versus the lowest tertile, p for trend = 0.05), whereas for MSI + stomach cancer cases, olive oil had also a protective effect, not statistically significant across tertiles (OR = 0.5, 95%CI 0.2 to 1.1, for the highest versus the lowest tertile, p for trend = 0.07) [54]. A protective effect of olive oil to stomach cancer was also detected in another Italian case-control study [55].

### Lung cancer

Evidence is scarce. In a hospital-based, case-control study of lung cancer in Italy (342 with primary lung cancer and 292 controls), use of olive oil found to offer a protection towards lung cancer (OR = 0.67, 95%CI 0.45 to 0.99) [56]. Prospective studies on the association of lung cancer with dietary fat were not taken into consideration in countries, such as USA and Norway, because monounsaturated fat intake was mainly from sources other than olive oil [57-59].

### Ovarian cancer

Evidence concerning olive oil in take in relation to ovarian cancer risk is limited; in two case-control studies, olive oil and monounsaturated fat (mainly olive oil) intake had a protective effect. Firstly, in Greece (189 epithelial ovarian cancer cases and 200 controls), an inverse relation of monounsaturated fat intake and risk for ovarian cancer was found (OR = 0.80, 95%CI 0.65 to 0.99 for 1SD increase in consumption on a daily basis) [60]. In a more recent case-control in Italy (1992 to 1999) 1031 cases and 2411 controls were included. After correction for multiple confounders, a reduced risk of ovarian cancer was observed for high intake of olive oil (OR = 0.68, 95%CI 0.50 to 0.93, for the highest quintile, compared with the lowest one), as well for higher intake of monounsaturated fat and oleic acid [61,62]. Other studies of diet and ovarian cancer were not taken into consideration, since the information was collected in non-Mediterranean countries [63,64].

### Endometrial cancer

In three epidemiologic studies, there is a suggestion of a protective effect. In a case-control in Switzerland and northern Italy (274 endometrial cancer cases and 572 controls), after correction for energy intake, more frequent consumption of olive oil was associated with a decreased risk for endometrial cancer, though results were not statistically significant (OR = 0.82 for highest vs. lowest tertile) [65,4]. Two hospital case-control studies have been published from Greece. In the first one (145 cases and 298 controls), the only statistically suggestive association was the inverse one with monounsaturated fats (OR = 0.74, 95%CI 0.54 to 1.03) [66]. In the second one (84 cases and 84 controls), a protective effect of added lipids, which in the Greek diet are primarily represented by olive oil, was highly suggestive [67].

### Pancreatic cancer

In two case-control studies, one yields inconclusive results for the association of monounsaturated lipids with pancreatic cancer, the second one shows a significant inverse association with olive oil. In a hospital case-control study conducted in Greece (181 cases and 181 hospital-181 hospital visitor controls), for a 1SD increase of the energy-adjusted residual of monounsaturated fat OR was 1.04 (95%CI 0.86 to 1.25) when compared with hospital controls and for 1SD increase in monounsaturated consumption OR was 0.97 (95%CI 0.80 to 1.17) compared to visitor controls, with no statistically significant association [68]. In northern Italy, in a case-control study conducted between 1983-1992 (362 cases with histologically confirmed, pancreatic cancer risk and 1502 controls), cancer risk was inversely associated with consumption of olive oil (OR = 0.58 for subsequent tertiles of intake) after allowance for sociodemographic factors and tobacco smoking (ORs were 0.76 for intermediate, and 0.60 for highest score of intake and the risk was significant) [69,70]. Monounsaturated fat intakes and oleic acid intakes in different countries than Mediterranean are not taken into consideration, since major sources of monounsaturated fat in North America is, for example, meat, and major sources of oleic acid in these countries could be canola, cod-liver, coconut, soybean, and almond oils [71-73].

### Bladder cancer

In a case-control study in Belgium (200 cases and 386 controls) results showed that there was a statistically significant inverse association between olive oil intake and bladder cancer consistent with a linear dose-response relationship: middle versus the lowest tertile (OR = 0.62, 95%CI 0.39 to 0.99) and the highest versus the lowest tertile (OR = 0.47, 95%CI 0.28 to 0.78; p-trend = 0.002) [74]. However, findings from another study conducted by Riboli and colleagues [75], showed that in a Spanish population with an average dietary pattern typical of Mediterranean populations, monounsaturated fat intake was associated with a moderate increase at risk, which disappeared after correction for saturated fat intake.

### Meta-analysis of the studies

**Figure 1:**
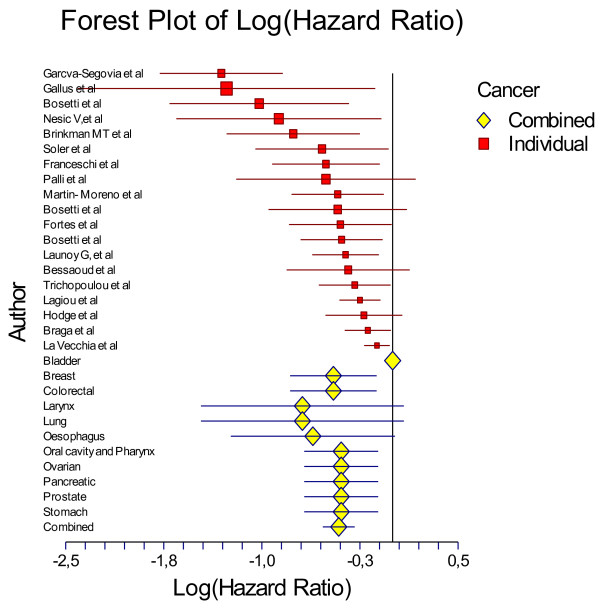
**Forest plot of studies that evaluated the association between olive oil intake on cancer development (data are presented as log Odds Ratios and the corresponding 95%CI)**.

As mentioned above for the meta-analysis 19 studies that evaluated solely olive oil intake were taken into account (Table 3). In total, data from 13800 cancer patients and 23340 controls were analysed in the present work. The combined effect of the highest percentile of olive oil intake compared with the lowest was highly significant (p < 0.001). Particularly, people in the highest group of olive oil consumption had 0.66-times lower odds of having any type of cancer (logOR = -0.41 95%CI -0.53 to -0.29) (Table 3) or 34% lower likelihood of having any type of cancer. The effect-equality test that was applied revealed that the study-specific effect size measures were heterogeneous (Cohran's Q = 47.52, I^2 ^= 62%, p = 0.0002), but all to the same protective direction (Figure 1).

**Table 3:** Meta-analysis of studies that evaluated the role of olive oil on cancer development.

	Combined effect log OR	95% CI for log OR; *p *for heterogeneity
All studies (n = 19)	-0.41	-0.53, -0.29; < 0.001
Type of cancer		
*Breast cancer (n = 5)*	-0.45	-0.78, -0.12; < 0.001
*Digestive (n = 8)*	-0.36	-0.50, -0.21; 0.16
*Other (n = 6)*	-0.41	-0.59,-0.23; 0.34
Region of origin		
*Mediterranean (n = 15)*	-0.43	-0.78, -0.12; 0.0002
*Non-Mediterranean (n = 4)*	-0.37	-0.62, -0.13; 0.12

Then, the studies were categorized by type of cancer, i.e., breast (n = 5 studies), digestive (i.e., colorectal, oral cavity, pharynx and oesophagus, pancreatic) (n = 8 studies) and other. Compared with low intake, high olive oil consumption was associated with lower odds of developing breast cancer (logOR = -0.45, 95%CI -0.78 to -0.12) and lower odds of developing a cancer of the digestive system (logOR = -0.36 95%CI -0.50 to -0.21) (Table 3). As regards the type of origin, in both Mediterranean and non-Mediterranean people that reported olive oil intake were less likely to have developed any type of cancer (Table 3). Heterogeneity of the effect-size measures was observed in studies performed in the Mediterranean region (*n *= 15, p = 0.0002), whereas no heterogeneity was observed in non-Mediterranean studies (*n *= 4, p = 0.12).

## Conclusion

Nutritional factors play a major role in cancer initiation and development [76]. The present systematic review and meta-analysis of observational studies revealed that, overall, olive oil consumption was associated with lower odds of cancer development. Most prominent results were observed for breast cancer and cancers of the digestive system, while the aforementioned relationship was similar to studies performed in Mediterranean as well as non-Mediterranean countries. Meta-analyses have several weaknesses, due to inherent biases and differences in study designs (different control for residual confounding, different socio-demographic and other lifestyle characteristics that may alter food habits), publication bias, etc [9]. The use of only case-control studies may also limit the interpretation of the results as causal. Moreover, in 2 studies no exact information about the effect size was reported in the retrieved abstract; however, the direction of the effect was similar to the other studies. Therefore, it could be speculated that the inability to include these studies in the meta-analysis would not alter the combined results. At this point it should also be noticed that although it is acceptable to consider studying the risk associated with extreme categories, such as the highest vs. lowest, the use of an ordered variable, like the effect sizes per incremental quintile would be preferable; however, the available results did not allow for such an analysis. Moreover, the analysis of studies was focused only on those that evaluated raw olive oil intake, in order to avoid the synergistic effect of cooking. Nevertheless, the large number of studies enrolled, makes the present work one of the few that systematically evaluated the cancer- monousaturated lipid (especially olive oil) associations.

Ecologic comparisons and meta-analysis of prospective cohort studies suggest that cancer morbidity and mortality are lower in Mediterranean countries, where olive oil represents a substantial fraction of dietary fat [77]. Prospective studies show evidence that higher degree of adherence to the Mediterranean diet is associated with a reduced mortality for cancer of all types, examined in one model[78]. According to a review, in Western countries, approximately 25% of the incidence of colorectal cancer, 15% of breast cancer, and 10% of prostate, pancreas, and endometrial cancer could be prevented if traditional Mediterranean dietary patterns were followed [79]. However, ecologic comparisons are characterized by complex forms of confounding, thus, are difficult to interpret [4], in addition, though the protective role of Mediterranean diet has been advocated by several studies, mutual confounding among food groups and nutrients, as plant foods and olive oil, have not been addressed in depth, and, it is probable that available Mediterranean dietary scales cannot capture all the interrelations among a multitude of foods consumed traditionally.

From ecologic comparison of 28 countries, 76% of the inter-country variation in colorectal cancer incidence rates could be attributed to three dietary factors: meat, fish and olive oil, in combination; meat and fish were found positively associated, whereas olive oil was negatively associated. The authors stated that olive oil could influence secondary bile acid patterns in the colon that, in turn, might influence polyamine metabolism in colonic cells reducing possibility to progression from normal mucosa to adenoma and, eventually, carcinoma [80]. Also, olive oil seems to be inversely associated with breast cancer risk, although there are no robust data to consider. Less experimental and epidemiologic evidence has been collected for the other types of cancer, such as gynecological, urological and respiratory cancers, and there are no available data for haemopoietic malignancies. As it is stated previously, most available studies till today are case-control ones and in the majority of them total energy intake is controlled for. Most of these studies are examining the role of a series of different nutrients and are not examining the specific role of added fats, monounsaturated fats (in Mediterranean countries) and in particular olive oil, to the above mentioned different types of cancer.

In addition, a recent meta-analysis of biomarkers of dietary fatty acid intake and risk of breast cancer concluded that there is no significant association between oleic acid and breast cancer risk in case-control studies, whereas in cohort studies there was significant higher risk for women with high levels of this fatty acid [81]. These papers referred were not taken into consideration, because it was not clear from the studies that oleic acid was deriving from olive oil only or from other dietary sources as well. Also, selection and nutrient-specific information biases are reported to be minimized in some of the studies taken into consideration, while others have also provided additional results after taking into consideration the mutual confounding by different food groups and nutrients, such as of olive oil and vegetables. Finally, though there is not enough data for skin cancer, it could be stated that due to the high concentrations of squalene in olive oil, which is transferred to the skin, olive oil intake could be searched for a protective effect against skin cancer [5].

Evidence to support that olive oil conveys protection against occurrence of different types of cancer necessitates more epidemiological studies, especially prospective ones, specifically designed to address these issues, which would all adjust for total energy intake. The ongoing and future well designed cohort studies will help to further examine the association and questions arising, such as, firstly if olive oil intake facilitates more vegetable intake, thus, maximising its beneficial effects to cancer prevention, and secondly, if the possible beneficial effects of olive oil are attributed to its monounsaturated content or to its other components, could be addressed [1,8,82]. Large enough and well conducted trials, both for countries with high as with low intake of olive oil, could be necessary, for the purpose of generalisability.

## Competing interests

The authors declare that they have no competing interests.

## Authors' contributions

TP designed and conducted research, wrote parts of the paper and had responsibility for its final content. RK, DH and MD wrote parts of the paper and had responsibility for its final content. DP analyzed data, wrote parts of the paper and had responsibility for its final content. All authors read and approved the final manuscript.
